# The Hidden Metabolic Threat: A Case of Fatty Acid Oxidation Disorder Masquerading as Viral Myocarditis

**DOI:** 10.7759/cureus.94734

**Published:** 2025-10-16

**Authors:** Hadhami Ben Turkia, Yusuf Hadi, Nader Khawaja

**Affiliations:** 1 Pediatrics, King Hamad University Hospital, Busaiteen, BHR; 2 Medicine, Royal College of Surgeons in Ireland, Manama, BHR; 3 Internal Medicine, King Hamad University Hospital, Busaiteen, BHR

**Keywords:** covid 19, dilated cardiomyopthy, fatty acid metabolism deficiencies, fatty acid oxidation disorder, inherited cardiomyopathy, long-chain fatty acid oxidation defect, mitochondrial trifunctional protein deficiency, viral-induced myocarditis

## Abstract

Fatty acid oxidation disorders (FAODs) are rare inherited metabolic diseases that can present with various features including cardiomyopathy, arrhythmias, hypoketotic hypoglycemia, and liver dysfunction. Their clinical and radiological manifestations may at times overlap with acquired conditions such as viral myocarditis and multisystem inflammatory syndrome in children (MIS-C), making timely diagnosis challenging. We describe a 10-month-old infant boy with undiagnosed long-chain FAOD (LC-FAOD) who presented with sudden cardiac arrest following a viral illness. The patient's family history was significant for cardiac disease and sudden death. The infant displayed early symptoms of cardiac dysfunction at three months and was misdiagnosed with COVID-19-related myocarditis at the age of six months. Despite initial cardiac recovery and metabolic stabilisation, the child remained comatose and succumbed to refractory respiratory distress syndrome. The initial workup identified metabolites suggestive of LC-FAOD and whole-exome sequencing (WES) identified a homozygous variant in HADHA gene, ultimately confirming the diagnosis of mitochondrial trifunctional protein deficiency (TFPD). Early recognition through newborn screening and genetic testing, coupled with timely initiation of targeted metabolic management, remains crucial to improving outcomes and preventing fatal consequences.

## Introduction

Mitochondrial β-oxidation of fatty acids (FA) is a crucial pathway to process energy for various organs during any hypercatabolic state. The very-long-chain acyl-CoA dehydrogenase (VLCAD) catalyzes the first step of mitochondrial β-oxidation of long-chain fatty acids (LC-FAO), which is then followed by the oxidation of 12 to 16 carbons facilitated by trifunctional protein (TFP), a multienzyme complex, which catalyses the latter three steps of the oxidation pathway [1]. TFP carries three important enzyme activities: long-chain enoyl-CoA hydratase (ECH), long-chain 3-hydroxyacyl-CoA dehydrogenase (LCHAD), and long-chain 3-ketoacyl-CoA thiolase (LCKAT) [2]. Dysfunction of one or more of these enzyme activities leads to either generalized TFP deficiency (TFPD), isolated LCHAD deficiency (LCHADD), or isolated LCKAT deficiency [2]. The HADHA gene (600890) encodes for ECH and LCHAD, and the HADHB gene (143450) encodes for LKAT. 

In individuals with LCHADD (OMIM: 609016), there is an isolated deficiency of LCHAD while deficiency of all three enzymes occurs in individuals with generalized TFPD (OMIM: 609105). TFPD is confirmed by the identification of deficiencies in all three TFP enzymatic activities in lymphocytes or skin fibroblasts [2]. TFPD is considered an extremely rare disease, with an incidence of 0.13 per 100,000 live births, diagnosed through newborn screening (NBS), as represented by a multi-national single study [3]. Among the North African region, the true incidence is unknown due to a lack of NBS programs and absence of published cases. Patients with TFPD and LCHADD display heterogeneous clinical phenotypes varying from early-onset life-threatening cardiomyopathy (CMP), arrhythmia, hypoketotic hypoglycemia, and liver failure, which may lead to death due to a late-onset form with myopathy, episodic rhabdomyolysis, peripheral neuropathy, and/or pigmentary retinopathy [2]. 

CMP in infants may arise from primary genetic defects or be secondary to inflammatory, neuromuscular, metabolic, mitochondrial, or syndromic disorders. Importantly, in patients presenting with severe cardiac dysfunction, distinguishing between inflammatory dilated CMP secondary to myocarditis and genetic dilated CMP can be challenging, yet is critical, as it carries significant prognostic and therapeutic implications. The EURObservational Research Programme (EORP) Cardiomyopathy and Myocarditis Registry, was a long-term perspective study that included 633 pediatric patients aged 1 to 18 years. Notably, those with coronary artery disease, hypertension, valve disease, or congenital heart disease (CHD) were excluded. It demonstrated that genetic etiologies represent a significant proportion of pediatric CMP. Non-syndromic CMP accounted for 72% of cases, inborn errors of metabolism for 6%, neuromuscular disorders for 7.7%, malformation syndromes for 11.8%, and chromosomal anomalies for 2.4% [4].

Cardiac involvement in fatty acid oxidation disorders (FAOD) can mimic other acquired or genetic CMP. The occurrence of the COVID-19 pandemic added an extra layer of difficulty in evaluating pediatric CMP. Cardiac manifestations of COVID-19 acute infection in children are relatively uncommon but may include myocarditis, pericarditis, pulmonary hypertension, and arrhythmias [5]. However, cardiovascular involvement is more common in multisystem inflammatory syndrome in children (MIS-C), a hyper-inflammatory condition occurring after COVID-19 infection, characterized by fever, elevated inflammatory markers, and at least two organ systems' involvement [6]. In a systematic review of 900 pediatric patients with MIS-C, 55% exhibited myocardial dysfunction or myocarditis, and 31.7% had pericardial effusion [7]. Patients with pre-existing CHD, genetic or metabolic CMP, or channelopathies represent a particularly high-risk population for myocardial dysfunction, arrhythmia, and sudden cardiac death in the COVID-19 infection or MIS-C. It is noteworthy to mention that sudden death occurs with an annual incidence of 2% to 3% in pediatric patients with dilated CMP [8].

There can be significant overlap in cardiac involvement seen in LCHADD/TFPD and in viral myocarditis. Both may present with cardiogenic shock or arrhythmias, creating a diagnostic dilemma and may lead to catastrophic outcomes. Herein, we report a fatal outcome in an infant with a delayed diagnosis of LCHADD/TFPD, initially misdiagnosed as COVID-19 myocarditis/MIS-C.

## Case presentation

We present the case of a 10-month-old Libyan boy who was brought to the ED after a sudden collapse at home. He was born at 36 weeks of gestation with antenatal complications of gestational diabetes and a birth weight of 2.4 kg. He is the fourth child to third-degree consanguineous parents. Family history is significant for an older sibling dying at the age of 20 months from suspected heart failure, another sibling with repaired CHD, and the sudden death of a maternal uncle at the age of 25 years.

Given the positive family history of cardiac disease, the patient underwent echocardiography at the age of two months, which was normal. At the age of 3.5 months, the mother noticed the child had poor feeding and activity. Later, both the 4.5-month-old infant and the mother developed flu-like symptoms during a local COVID-19 outbreak, at which no medical attention was sought. At the age of six months, the child was admitted to another medical facility for poor feeding and activity, excessive sweating, and irritability. He was diagnosed with heart failure, with a reduced ejection fraction (EF) of 21%. As COVID-19 serology was positive, the child was diagnosed with MIS-C myocarditis and received intravenous immunoglobulins with anti-heart failure medications. Follow-up echocardiography showed improvement in cardiac function.

A visit to a cardiac center abroad at the age of eight months revealed mildly impaired systolic function with a marginally dilated left ventricle and normal diastolic function on echocardiography. Cardiac MRI showed EF of 57%, subsiding myocarditis with evidence of residual edema, normal left ventricular function, and a mild pericardial effusion. The same diagnosis of resolving myocarditis/dilated CMP, probably of infective origin, was given. One month later, the patient was admitted in the same local facility for bronchiolitis, and a drop in EF to 50% was noted. The patient did not require intensive care during his stay. 

**Figure 1:**
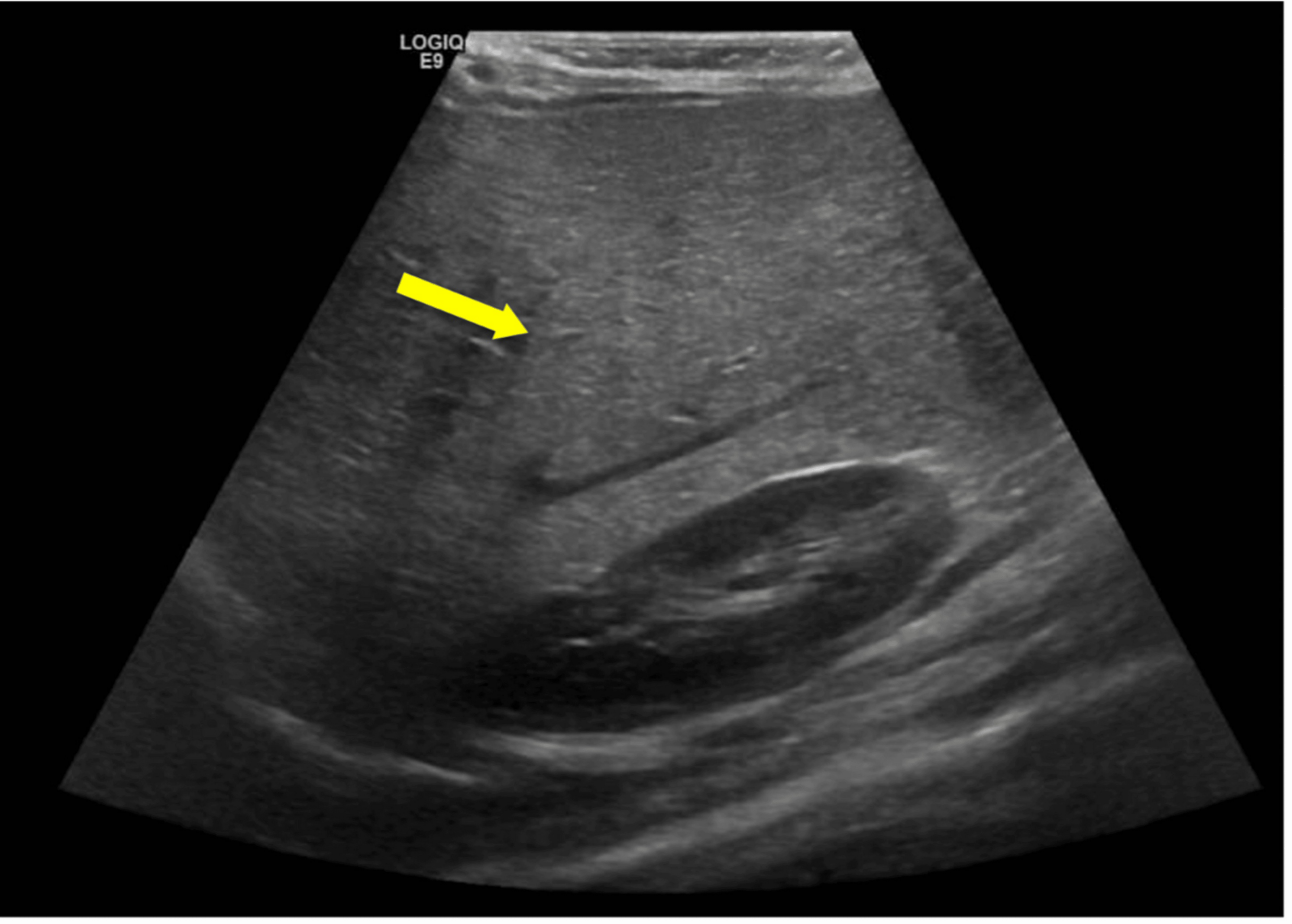
Abdominal ultrasonography image showing increased echogenicity of the liver compared to surrounding structures like the kidney, as indicated by the arrow, supporting the presence of a fatty liver.

Twenty-four hours after his discharge, the patient collapsed at home. Upon admission to our ED, the patient was in asystole. Return of spontaneous circulation (ROSC) was achieved after 30 minutes of CPR. Assessment of the patient post-ROSC revealed the patient to be in an unconscious state, with a Glasgow Coma Scale (GCS) score of three and slow-reacting pupils bilaterally, 2 mm in size. Additionally, on abdominal examination, an enlarged firm liver 6 cm below the costal margin was present. Blood gas analysis at admission revealed a low serum pH of 6.809, pCO_2_ of 83.5 mmHg, HCO_3_ of 13 mmol/L, and a raised lactate level of 17.7 mmol/L. Capillary glucose of 0.9 mmol/L and blood ketones of 0.4 mmol were also reported. The provisional differential diagnoses at that time included acute decompensation of post-COVID-19 myocarditis/MIS-C versus metabolic CMP, given the strong family history of cardiac disease, hepatomegaly, and hypoketotic hypoglycemia. Therefore, the patient was started on ventilatory support and inotropes. Initial laboratory tests showed deranged liver function tests, coagulation profile, creatine phosphokinase (CPK), and cardiac enzymes. Acute phase reactants were elevated. COVID-19 antibodies were positive. The laboratory results are summarized in Table 1. Initial bedside echocardiography showed EF of 20-25%, which subsequently increased to 40% following inotropic support. Ultrasonography of the abdomen revealed an enlarged fatty liver with an edematous gallbladder (Figure 1).

**Table 1:** Key laboratory findings including hematological, biochemical, inflammatory, cardiac, and virological results.

Parameter	Result	Reference Range
Complete Blood Count (CBC)		
Hemoglobin	11.4 g/dL	10.5-13.5 g/dL
Total Count	5.8×10⁹/L	6.0-17.5 ×10⁹/L
Platelets	356×10⁹/L	150-450 ×10⁹/L
Neutrophil Percentage	46%	20-40%
Cardiac Markers		
Troponin I	130 ng/L	20-60 ng/L
Lactate dehydrogenase (LDH)	3752 U/L	225-600 U/L
B-type natriuretic peptide (BNP)	144 pg/mL	5-100 pg/mL
Creatine phosphokinase (CPK)	14,952 U/L	50-400 U/L
Creatine kinase-myocardial band (CK-MB)	133 ng/mL	<5 ng/mL
Coagulation Profile		
Prothrombin time (PT)	26.5 seconds	10.5-13.5 seconds
International normalized ratio (INR)	2.28	0.8-1.2
D-dimer	80 mg/L FEU	<0.5 mg/L FEU
Liver Function Tests		
Aspartate aminotransferase (AST)	2888 U/L	25-75 U/L
Alanine aminotransferase (ALT)	1340 U/L	10-45 U/L
Direct bilirubin	5.7 µmol/L	<5.1 µmol/L
Albumin	2.82 g/dL	3.5-5.2 g/dL
Renal Function Tests		
Urea	9.6 mmol/L	1.8-5.4 mmol/L
Creatinine	44.5 µmol/L	18-35 µmol/L
SARS-CoV-2 Microbiology		
N (Nucleocapsid) Protein Antibodies	Reactive	Non-reactive
S (Spike) Protein Antibodies	430 U/mL	<0.8 U/mL
Neutralizing Antibodies (nAbs)	92.60%	<30%
Polymerase chain reaction (PCR)	Negative	Negative
Inflammatory Markers		
C-reactive protein (CRP)	58 mg/dL	<5 mg/L
Procalcitonin (PCT)	5.52 ng/mL	<0.1 ng/mL
Ferritin	5438 ng/mL	10-100 ng/mL
Interleukin 6 (IL-6)	523.2 pg/mL	<7 pg/mL
Carnitine Profile		
Free Carnitine (C0)	5.5 umol/l	>8.2 umol/l
Hydroxypalmitoylcarnitine (C16OH)	0.30 umol/l	0.13 umol/l
3-Hydroxyoleoylcarnitine (C18:1OH)	0.38 umol/l	0.20 umol/l
Hydroxystearoy|carnitine (C18OH)	0.15 umol/l	0.10 umol/l
Others		
Respiratory Panel	Positive for respiratory syncytial virus (RSV) and parainfluenza virus 4	Negative

A metabolic workup, including tandem mass spectrometry (TMS), serum total and free carnitine, serum acyl-carnitines profile, urinary organic acids, was conducted and was suggestive of LCHADD/TFPD. Serum acyl-carnitines profile showed high levels of hydroxypalmitoylcarnitine (C16:OH), 3-hydroxyoleoylcarnitine (C18:1OH), hydroxystearoylcarnitine (C18OH), and low free carnitine level. Moreover, significant increases in adipic acid, lactic acid, pyruvic acid, suberic acid, 5-hydroxyphenyllactate, and 4-hydroxyphenylpyruvate were detected in the urine. The patient received respiratory and hemodynamic support and was started on broad-spectrum antibiotics, IV fluid dextrose with a glucose infusion rate (GIR) of 8 mg/kg/min, carnitine, biotin, and thiamine. In view of possible MIS-C, intravenous immunoglobulins (2 g/kg) and pulse methylprednisolone were initiated. After 48 hours, the patient was started on a monogen formula. This low-fat formula is low in long-chain triglycerides and high in medium-chain triglycerides (MCT) and used to bypass the enzymatic block.

Cardiac markers normalized after one week of initiating treatment and continued to decline (Table 2). Repeated echocardiography after 10 days showed EF of 58%; thus, inotropes were stopped. Repeated TMS revealed normalized acyl-carnitines. His sensorium remained waxing and waning with a maximum GCS of E3-VET-M4. Later, the child had features of severe hypoxic ischemic brain injury, evident by conjugate eye movements with intermittent posturing. The EEG was abnormal and showed a continuous slowing of brain activity while the patient was awake, likely indicating generalized cortical dysfunction. Also, MRI of the brain was normal.

**Table 2:** Trend of cardiac markers after initiating metabolic treatment

Cardiac Marker	Level at the Beginning of Treatment	Level After 1 Week of Treatment	Level After 2 Weeks of Treatment	Reference Range
B-type natriuretic peptide (BNP)	144.76 pg/mL	84.60 pg/mL	84.37 pg/mL	5-100 pg/mL
Tropinin I	130.00 ng/L	24.00 ng/L	19.90 ng/L	20-60 ng/L
Creatine kinase-myocardial band (CK-MB)	34.70 ng/mL	2.80 ng/mL	20.10 ng/mL	<5 ng/mL
Creatine kinase (CK)	14,952.40 U/L	67.10 U/L	168.4 U/L	50-400 U/L

In view of failed extubation and poor sensorium, the patient underwent a tracheostomy. During his hospital stay, he developed ventilator-associated pneumonia and had further cardiac dysfunction secondary to sepsis and metabolic decompensation. He later died from refractory acute respiratory distress syndrome (ARDS).

Whole-exome genome sequencing revealed a homozygous variant in the HADHA gene c.2026C>T p.(Arg676Cys) on exon 19, which was classified as a variant of unknown significance (Class 3), according to the American College of Medical Genetics and Genomics (ACMG). Whole-exome sequencing was also performed on both parents and the patient's sister, and the same variant heterozygosity was present in both parents.

## Discussion

High-energy demand organs like the brain, heart, skeletal muscle, and liver all rely on fatty acid β-oxidation (FAO) as a crucial source of energy. CMP and arrhythmias are well-recognized complications of several LC-FAO disorders (LC-FAOD), most commonly VLCAD deficiency (VLCADD), LCHADD, and TFPD. VLCADD and LCHADD are particularly associated with hypertrophic CMP, whereas TFPD can present either with hypertrophic or dilated CMP [9]. The underlying pathophysiology behind such complications is linked to deficiencies in energy substrates to high-energy demand organs and the subsequent accumulation of toxic, arrhythmogenic long-chain acylcarnitines [10]. Various stresses, such as fasting, fever, and hypercatabolic state, trigger metabolic decompensation. 

The infant had progressive symptoms of CMP at three months of age, preceding his COVID-19 infection, and developed cardiac failure at six months. In view of positive COVID-19 antibodies, he was misdiagnosed with COVID-19-related myocarditis until he suddenly collapsed at 10 months, following a viral illness. Unfortunately, no metabolic workup was performed initially despite a family history of cardiac disease and sudden death. Indeed, patients with CHD and genetic CMP are at additional risk of myocardial dysfunction, arrhythmia, and sudden cardiac death during COVID-19 infection [11].

Other than the underlying metabolic condition, the cardiac dysfunction in our patient could have arisen from multiple factors, including direct viral myocardial injury and the hyperinflammatory, immune-mediated cytokine storm of MIS-C. The temporary improvement in left ventricular fraction may suggest the potential benefits of immunoglobulin therapy as an immunomodulator. The delay in complete recovery and the secondary deterioration allude to the fact that the patient developed COVID-19 myocarditis, which decompensated his underlying metabolic CMP. Although most patients with COVID-19 myocarditis have a complete recovery in two to four weeks [12], 20%-30% of patients progress to dilated CMP, making the distinction between myocarditis and genetic CMP challenging [13]. 

From this standpoint, Gran et al. conducted a retrospective descriptive study to identify patterns that differentiate patients with acute myocarditis from those with dilated genetic CMP [13]. The study encompassed 27 pediatric patients with new-onset heart failure and a left ventricular ejection fraction <35%, of whom 18 were diagnosed with acute myocarditis and nine with dilated genetic CMP. An endomyocardial biopsy (EMB) was performed in 85% (23/27) of the patients. The findings were more consistent with myocarditis rather than genetic CMP and included a fulminant or acute presentation, higher levels of cardiac enzyme elevation, a lower left ventricular dimension z-score, increased ventricular wall thickness, and evidence of edema on EMB. However, 78% of patients with genetic CMP also demonstrated inflammation on EMB, fulfilling the diagnostic criteria for inflammatory CMP. The authors noted that these patients likely had concurrent acute myocarditis. On the other hand, because the yield of genetic testing is low in certain CMP subtypes, a genetic cause might have been missed in the myocarditis group.

The MRI findings of residual edema and pericardial effusion are consistent with direct viral injury, although pericardial effusion could occur independently as a manifestation of FAOD. Interestingly, massive pericardial effusion and hypertrophic CMP were the initial presentation of VLCAD deficiency in a five-month-old baby that improved only after the administration of MCT formula [14].

Metabolic causes of CMP should be considered in the differential diagnosis of any CMP of childhood, especially in infants, patients with multi-system involvement, certain CMP phenotypes (hypertrophic, dilated or left ventricular noncompaction), a rapidly progressive course, or positive family history. Metabolic workup, including TMS, serum total and free carnitine, serum acylcarnitine profile and urine organic acids, should be collected during the acute metabolic crisis. Genetic testing should be performed even if no obvious metabolic abnormalities are detected. In the unfortunate event of death, collecting blood or skin samples for DNA extraction may be useful for genetic studies. Family relatives should also be screened if a causative variant is identified in the proband.

The absence of neurological recovery in our patient could be explained by prolonged cardiac arrest and severe acute decompensation of FAOD, manifesting as metabolic encephalopathy. Prolonged coma or irreversible neurological injury following treatment of acute decompensation is a rare but recognized outcome of FAODs. Though most reported cases that receive prompt treatment lead to full neurological recovery, the risk of persistent coma or serious neurological injury is higher when there is a delay in the management of metabolic crises, profound hypoglycemia, hepatic failure, or cardiac dysfunction [15]. All of these factors were present in our patient.

Cardiac arrhythmia was the leading cause of death in our patient, given that the left ventricular ejection fraction (LVEF) was 55% just 24 hours prior to his cardiac arrest. Such arrhythmias often occur suddenly at home, where heart rate and rhythm monitoring are unfeasible. Similarly, a fatal acute CMP developed during the course of a COVID-19 infection in a patient with LCHADD/TFPD despite normal echocardiographic findings prior to the infection [16].

Among 500 cases of sudden infant death syndrome evaluated through post-mortem metabolic autopsy over a period of five years, Rinaldo et al. detected 44 cases of FAOD, accounting for 8.8% [17]. Of those cases, 10 (23%) died within the first week of life. Their investigations unveiled a case of carnitine palmitoyltransferase II deficiency and four cases of FAO-related gene variants.

A 2003 case-based series detailing 21 patients diagnosed with TFPD was conducted in the UK and the Netherlands [18]. The median age of presentation was three months, with nearly half (48%) presenting during the neonatal period. CMP was detected prenatally in two patients, suggesting the prominent role of FAO in fetal myocardial tissue. The overall reported mortality was 76%. In that cohort, two clinical phenotypes were identified: 43% (9/21) had an early-onset, rapidly progressive course and died of cardiac failure within eight weeks. Meanwhile, 57% (12/21) followed a more insidious trajectory, similar to our patient, characterized by feeding difficulties, failure to thrive, and hypotonia, with a higher incidence of CMP (73%). In the latter group, 58% (7/12) died due to progressive CMP, metabolic decompensation, or arrhythmias.

In a French cohort of 187 patients diagnosed with FAOD below the age of six years, the overall mortality rate was 50%, with life-threatening events occurring more frequently in the first two months of life [19]. CMP and arrhythmias occurred either in combination or as isolated outcomes. Moreover, 62% of all arrhythmias encountered in the study were ventricular tachycardia. Hepatomegaly and liver steatosis observed in 90% and 88% of patients, respectively, were also present in our case. Liver steatosis, often accompanied by fibrosis, occurs due to the accumulation of fatty acid esters and is considered a characteristic clue of FAOD. Hypoketotic hypoglycemia, hyperammonemia, hyperlactacidemia, and impaired liver function were the most associated biochemical abnormalities in the cohort.

To date, 125 different mutations in the HADHA gene have been reported, including the c.2026C>T p.(Arg676Cys) variant identified in our patient. The only reported case with the same variant as our patient presented with a "cardiac phenotype", but insufficient details were provided in the study [20]. The genotype-phenotype correlation for HADHA mutations remains unclear. In the absence of the common LCHADD variant (c.1528G>C) or enzymatic assays for all three components of the TFP complex, it is often difficult to clinically distinguish between LCHADD and complete TFPD. Additionally, compound heterozygosity (the common variant and one other rare mutation) is frequent in many ethnicities outside Northern Europe and can be associated with either LCHADD or TFPD [19]. Nonetheless, this has no management implications as both conditions have the same treatment. Several case reports suggest that complete TFPD typically presents earlier and is associated with more severe cardiac disease. 

Acute management of FAOD metabolic crises involves administering 10% dextrose at 1.5 to two times the required maintenance rate [2]. MCT oil should be initiated as soon as enteral feeding is tolerated. L-carnitine supplementation is useful in documented carnitine deficiencies, though its use in the absence of such deficiency remains controversial. A thorough cardiac evaluation using echocardiogram and ECG is essential due to the possibility of arrhythmias and CMP arising in metabolic crises. In order to screen for hepatic dysfunction and myopathy, regular monitoring of liver function tests and creatinine kinase levels is imperative. Meanwhile, long-term management involves avoidance of fasting altogether, adherence to a low-fat, high-carbohydrate diet, MCT oil supplementation to provide 15% to 25% of the daily energy requirements, and carnitine supplementation when indicated. Triheptanoin (C7), an anaplerotic odd-chain MCT, was recently approved in the treatment of LC-FAOD and is proven to reduce the incidence of hospitalizations, rhabdomyolysis, CMP, and hypoglycemia [2]. Importantly, CMP may resolve completely if adequate metabolic control is achieved.

Screening newborns for FAODs in developed countries has notably reduced morbidity and mortality. However, neonates can still decompensate before the results of the NBS are acquired; hence, physicians should have a high index of suspicion for such diseases, particularly in neonates with hypotonia, cardiac dysfunction, high muscle enzymes, or hypoketotic hypoglycemia. In our country, there is still no standardized national NBS program for all metabolic disorders. This case is a reminder of the importance of adding TMS in NBS programs, as it could have unravelled the underlying disease early on and prevented a fatal outcome.

## Conclusions

FAOD represents a rare but critical entity behind cardiomyopathy and metabolic decompensation occurring in the pediatric population. Their clinical presentation can overlap with more common acquired conditions, often leading to a delay in diagnosis. In general, metabolic causes, including LC-FAOD, should be considered in the differential diagnosis of any cardiomyopathy of childhood, and physician awareness of such disorders is extremely crucial. Family screening of siblings and referral to a genetic counselor is also essential, as this disease can present at any age. Early detection aids in implementing appropriate management, which can prevent acute decompensation and mortality. This case highlights the necessity of prompt detection of metabolic disorders like FAOD, particularly in the context of viral infections.
